# Supplementary material for: Towards unified AI-driven fracture mechanics: the extended deep energy method (XDEM)
Source: Nat Commun. 2026 Aug 15;17:8492. doi: 10.1038/s41467-026-76748-1 (PMC13484471; doi:10.1038/s41467-026-76748-1)
Supplement: Supplementary file 3 [file 41467_2026_76748_MOESM3_ESM.zip › pic/Results/Crack_propagation/inclusion/load_displacement_comparison.pdf]

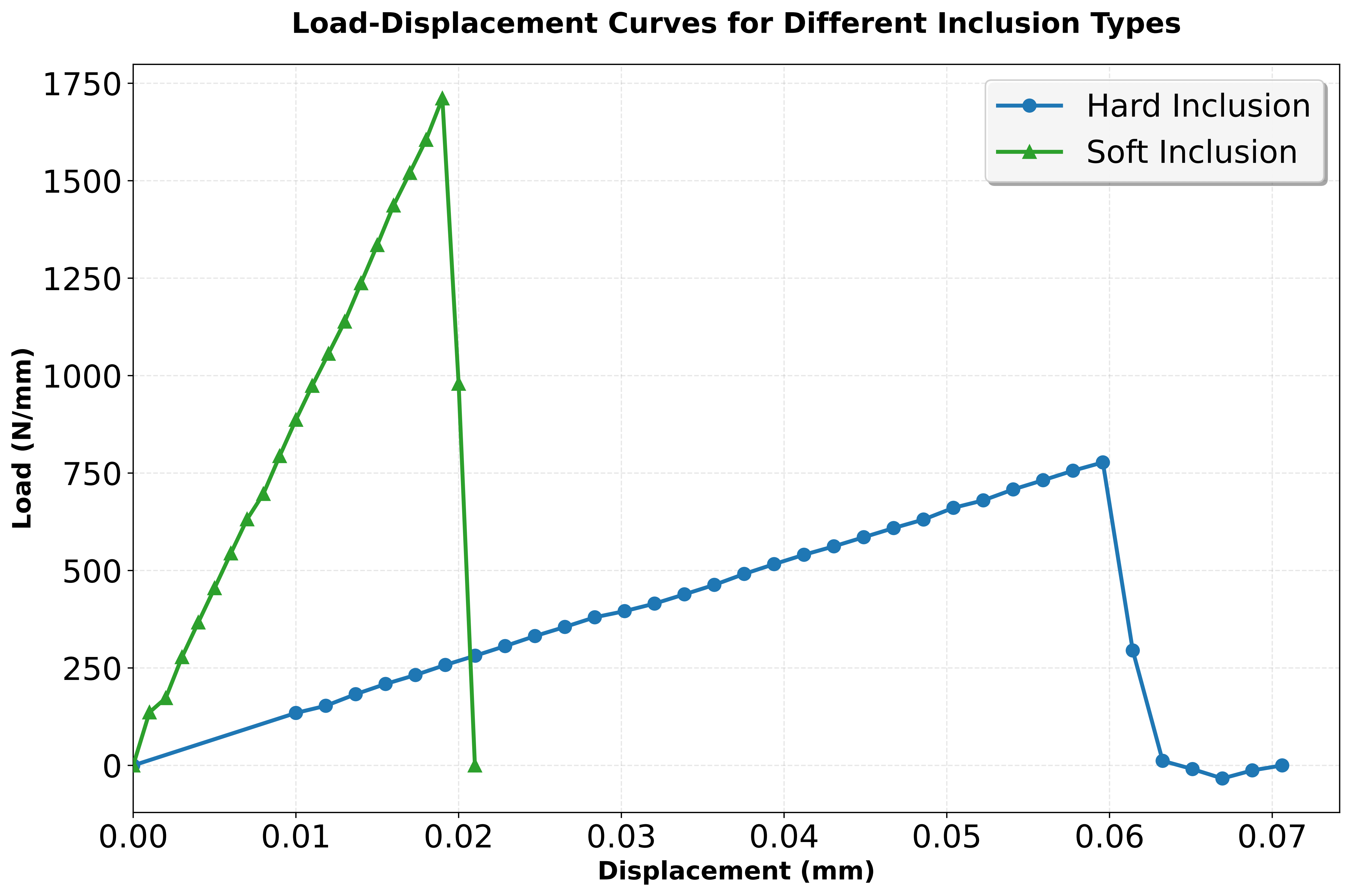

Load-Displacement Curves for Different Inclusion Types
Hard Inclusion
Soft Inclusion
Load (N/mm)
Displacement (mm)
0
250
500
750
1000
1250
1500
1750
0.00
0.01
0.02
0.03
0.04
0.05
0.06
0.07
